# Current Challenges in Hematology: Awareness, Prevention, Equity

**DOI:** 10.3389/fonc.2021.653020

**Published:** 2021-04-27

**Authors:** Dominic Kaye, Alessandro Isidori

**Affiliations:** ^1^ Frontiers in Oncology, Frontiers Media SA, Lausanne, Switzerland; ^2^ Hematology and Stem Cell Transplantation, AORMN Hospital, Pesaro, Italy

**Keywords:** hematologic malignancies, health equity, cancer care, access to cancer services, cancer awareness, cancer prevention

## Introduction

Hematologic malignancies make up approximately 10% of all cancer types in the USA, and the management of patients suffering from hematologic malignancies has dramatically changed over the last 20 years (1). Death rates have reduced across the various malignancy types, and rapidly fatal diseases, such as chronic myeloid leukemia, have become curable thanks to therapies like Imatinib Mesylate, occasionally termed an oral ‘magic bullet’ (1–3). Pathologies ignored by most, with little or no therapeutic possibilities, based only on conventional chemotherapy, have captured the spotlight: chronic lymphocytic leukemia, C(LL)inderella, very recently became a star, with numerous ongoing clinical trials and several novel agents approved (4, 5). Another case in point is the outcome of multiple myeloma (MM), the second most frequent hematologic malignancy type after non-Hodgkin lymphoma (NHL), has significantly improved in recent years, again thanks to the introduction of novel therapeutic agents (6). A better understanding of disease heterogeneity, together with the discovery of novel targeted agents to be used in combination with chemotherapy, has also significantly improved the prognosis of acute myeloid leukemia (AML), the big bad wolf for any hematologist (7). Lastly, the advent of Chimeric Antigen Receptor T (CAR-T) Cells has brought upon similar advances and positive outcomes in a number of patients, and as of February 2021, there are four CAR-T Cell therapies approved by the FDA for use in patients, and numerous additional ongoing clinical trials. CAR-T Cells are a novel personalized cancer therapy which acts directly on the immune system of patients, making it able to recognize and destroy tumor cells, and has granted the opportunity to treat advanced forms of hematological malignancies in resistant and relapsed patients, and to survive (8, 9).

What challenges does a hematologist face in 2021, if any? As a matter of fact, all that glitters is not gold. This year’s World Cancer Day is focused on awareness, prevention and equity. When thinking about it, it is not strange that these are the themes that were selected this year. Let’s explore why.

## Awareness

Wikipedia defines awareness as “the state of being conscious of something. More specifically, is the ability to directly know and perceive, to feel, or to be cognizant of events” (10). When applied to patients, awareness is the state of being conscious of the disease and its symptoms. It is therefore necessary to ensure that screening, early detection, and education surrounding the disease is further increased. A lack of awareness can be attributed to a number of reasons. An inability to access information, and more importantly accurate information, is one key reason for a lack of awareness. This, unfortunately, can often be made harder through the presence of stigma associated to cancer, which can prevent individuals from taking preventative action, or even receiving medical check-ups, out of fear. A case in point is, according to an UICC report, only 36% of people surveyed across Africa view cancer as a major health issue, and 25% believe that cancer has no cure (11). If people are aware of a disease and its symptoms, they are more likely to act in order to prevent it from happening to them, through means such as participating in screening programs, regularly checking their health status, and visiting their doctor when they experience symptoms. As a result of this aforementioned lack of awareness, people may come to hospitals when their disease has degenerated, or reached an advanced stage, resulting in lower possibilities of receiving effective treatment and curing their tumors. A case in point is in Indonesia, where approximately 70% of cancer patients visit a physician once their cancer is at a late stage, thus reducing their overall chance of survival (11).

Lack of awareness may not only worsen clinical outcome, but it can also be divisive in society and affect quality of life. As a hypothetical example, a young child with leukemia (in remission) may not be invited to attend a peer’s birthday party as the hosts are scared to invite a leukemic patient, stemming from the stigma associated to condition, immunodeficiency, risk of bleeding, and in some cases, there can even be a misconception that the cancer itself is contagious.

Why is there a push to increase awareness surrounding leukemia, lymphoma, myeloma, and other hematological malignancies? The best opportunity for awareness campaigns is in changing attitudes and behaviors toward cancer prevention, screening, and early detection. Unfortunately, these strategies are very limited in leukemia, lymphoma, and myeloma, given the rapid and aggressive course of these diseases. However, awareness and education can initiate a cascade. Increased awareness and education lead to further support, which in turn allows for more research. And research leads to new and better treatments. With this aim, September was selected to be Blood Cancer Awareness Month each year, in order to raise funds for research and patient support organizations involved in hematological malignancies.

Unsurprisingly, a Google web search of ‘cancer awareness month’ is dominated by results related to “Pink October”, which pertains to breast cancer awareness. Breast Cancer Awareness month, famously associated with the color pink, receives huge amounts of publicity, with numerous articles being published in popular media outlets rather than scientific journals. A PubMed search on ‘cancer awareness months’ identifies even fewer publications, and once again the results are predominantly related by breast cancer. Jacobsen and Jacobsen (12) investigated a potential relationship between the Breast Cancer Awareness Month initiative, and the number of subsequent breast cancer diagnoses in the following month, November, looking at years prior to, and following the introduction of the initiative (using SEER data to investigate such numbers). The authors demonstrated that between 1973 and 2005 there were some evident “spikes” in diagnoses in the month following Breast Cancer Awareness month, November (12). Two of these apparent increases in diagnoses could in fact be attributed to an overall increased awareness related to breast cancer diagnoses in females, as opposed to, for an example, and advance in diagnostic methods. In particular, there was a clear spike in November diagnoses in the mid-1990s, in the 3 years following the initial introduction of Breast Cancer Awareness Month. However, this increased trend did not continue in subsequent years (12). Breast cancer screening rates have significantly increased in the last 30 years, and the distribution of breast cancer diagnoses throughout the year has become more consistent and uniform, without any additional “November spikes”. In brief, the introduction of a dedicated awareness month initiative was initially highly effective, and it is plausible that the importance of mammography was recognized thanks to the effect of a short-term awareness campaign, bringing further attention to breast cancer.

Awareness is extremely important also for patients who have endured and survived cancer, in particular the pediatric population, who are exposed to late effects of treatment. Such effects can have varied onset – months or even years after treatment, and are heterogenous in nature spanning from physical, cognitive, or psychological. A recent systematic literature review, with input from >20 different organizations, resulted in the creation of LEAP^3^ AHEAD (Late Effects Awareness for Patients, Physicians and the Public; Advancing Health and Eliminating All Disparities), a multi-dimensional website centered on late effects (13). This is the first interactive, international website dedicated to acute lymphoblastic leukemia childhood cancer survivors and families, as well as physicians. It was developed with the aim to increase awareness about risks, detection, diagnosis, treatment, and prevention of medical and psychological late effects (13). Awareness and prevention are two sides of the same coin.

## Prevention

Wikipedia defines prevention, referring to healthcare, as “preventive healthcare”: measures to prevent diseases or injuries rather than curing them or treating their symptoms (14). Although great strides have been made to cure several types of cancer, cancer remains the second leading cause of death worldwide. Instead of waiting for state-of-the-art treatment breakthroughs, individuals can take action to protect themselves. Examples of such actions include participating in early screening, as well as following some general recommendations and tips to prevent cancer development. Such recommendations often form the basis of national initiatives aimed at curbing cancer rates. Early diagnosis is important; however, reducing the risk of getting cancer is even more important. Avoiding tobacco in all its forms, consuming a balanced diet, actively avoiding obesity, exercising regularly, consuming alcohol with moderation, and making quality sleep a priority, are easy and apparent ways to help prevent cancer, even if many people do not strictly follow them. Getting vaccinated, protecting our body from the sun, avoiding risky behavior such as unnecessary exposure to radiation or to industrial and environmental toxins are important things to keep in mind, especially in preventing hematologic neoplasms.

In order to stress the importance of our behavior in preventing cancer development, we appoint several examples. 1. The bone marrow is one of the most radiosensitive organs, and there is clear evidence that the risk of developing AML, acute lymphoblastic leukemia (ALL), and myelodysplastic syndromes (MDS) are higher after exposure to moderate-to-high doses (>0.5 Sv) of ionizing radiation (15). Moreover, exceptionally high relative risks of deferred leukemia have been described following radiation exposure in childhood (15, 16). The majority of exposures, however, are typically at low doses as they result from natural background radiation, diagnostic medical tests, or occupational exposure. Accordingly, evaluating risks in the low dose range is critical for radiation-protection purposes, especially in children. Interestingly, recent data suggest that even exposure to low-dose radiation <100 mSv, or even <50Sv during childhood, may be associated with increased risk of childhood ALL and subsequent MDS/AML (15, 16). This finding is particularly relevant as computerized tomography is a common source of low dose radiation. *Nikkilä et al.* demonstrated that cumulative red bone marrow dose from computed tomography (CT) scans showed an excess odds ratio of 0.13 (95% confidence interval: 0.02 - 0.26) per mGy. In other words, whilst CT may be a helpful diagnostic tool, it is on the other hand a source of low-dose radiation which can subsequently result in an increased risk of leukemia. 2. There is abundant evidence about the association between viral infection and lymphoma development. Epstein-Barr Virus infection is responsible for African Burkitt lymphoma, and increases the risk of both Hodgkin disease and follicular lymphoma (17, 18). Furthermore, Hepatitis B and C infections are similarly associated with an increased risk of non-Hodgkin lymphoma (19), with an additional risk increase in patients with concomitant HIV infection (20). *Fabula docet:* prevention is (at least) half of the cure, and the higher is the awareness, the better the prevention.

## Equity

Equity can be a difficult concept to comprehend as the term itself has multiple definitions depending on the context, ranging from a financial setting, to in this case, the public health setting. Health equity, in its simplest form, means giving patients the care they need when they need it. The US Institute of Medicine indicates, health equity is “providing care that does not vary in quality because of personal characteristics such as gender, ethnicity, geographic location, and socioeconomic status” (21). Accordingly, and before looking at health equity in cancer, it is quite clear that awareness, prevention, and equity all go hand in hand. Higher rates of chronic and expensive illnesses, together with high rates of uninsured people among lower socioeconomic and minority populations, result in a greater reliance on emergency services, higher treatment costs, and, finally, a financial strain on providers and government programs. As an example, in rural parts of the USA, employer insurance coverage is lower than in comparable urban areas (22). Similarly, lower socioeconomic status among cancer patients has been seen in some cases to be associated with an elevated risk of suicide (23). We know that preventive medicine and early interventions are proven means to saving money and lives.

Returning back to cancer, the gains that have been made in the reduction of cancer incidence and mortality are unfortunately not shared by everyone. Disparities still exist with regards to ethnicity, gender, race, socioeconomic status, and geography (24). As an example, a review of global access to cancer medicines in 2020 showed a marked difference in the availability of cancer medicines between countries of varying income levels. Only 10% of countries around the world listed all 25 cancer medications that appear on the World Health Organizations Model *Lists of Essential Medicines*. In contrast, on average, only 9 listed medicines were available in low-income nations (25).

In 2008, the National Institutes of Health Centers for Population Health and Health Disparities published a framework to elucidate the ecological determinants of disparities in cancer incidence, morbidity, and mortality (26). Interventions to eliminate disparities may be aimed at the multilevel factors, required to achieve improved cancer outcomes. Health systems are paying attention to disparities in the quality of their care and seeking remedies as healthcare costs rise, and consumers demand action.

In conclusion, awareness, prevention, and equity are still hot topics, even in 2021, as people still die of cancer in 2021, still due to a lack of awareness, prevention, equity. This is why it is so important that the 2021 World Cancer Day is dedicated to awareness, prevention and equity

## Author Contributions

Both authors equally contributed to the writing of this manuscript.

## Disclaimer

Dominic Kaye is an employee of the Frontiers in Oncology Editorial Office.

## Conflict of Interest

Since [2018], the co-author [DK] has been employed by Frontiers Media SA. [DK] declared his/her affiliation with Frontiers, and the handling Editor states that the process nevertheless met the standards of a fair and objective review.
